# The role of *Chlamydia trachomatis* in preterm delivery: a case-control study in Besat Hospital, Sanandaj, Iran (2018–2019)

**DOI:** 10.18502/ijm.v12i4.3936

**Published:** 2020-08

**Authors:** Seyedeh Jahan Ahmadi, Fariba Farhadifar, Seyedeh Reyhaneh Yousefi Sharami, Shamsi Zare, Masomeh Rezaei, Nasrin Soofizadeh, Erfan Babaei, Ghobad Moradi, Amjad Ahmadi

**Affiliations:** 1 Department of Obstetrics & Gynecology, School of Medicine, Kurdistan University of Medical Sciences, Sanandaj, Iran; 2 Social Determinants of Health Research Center, Research Institute for Health Development, Kurdistan University of Medical Sciences, Sanandaj, Iran; 3 Department of Immunology, School of Medicine, Kurdistan University of Medical Sciences, Sanandaj, Iran; 4 Department of Microbiology, School of Medicine, Kurdistan University of Medical Sciences, Sanandaj, Iran; 5 Cellular and Molecular Research Center, Research Institute for Health Development, Kurdistan University of Medical Sciences, Sanandaj, Iran

**Keywords:** *Chlamydia trachomatis*, Preterm delivery, Women, Iran

## Abstract

**Background and Objectives:**

Preterm delivery is an important subject in gynecology, obstetrics and pediatrics. It is defined as regular uterine contractions every five to eight minutes or less, lasting for 30 seconds. It is associated with progressive changes in the cervix, resulting in delivery after 22 weeks and before 37 weeks of gestation. This study aimed to evaluate the role of *Chlamydia trachomatis* infection in women with preterm delivery.

**Materials and Methods:**

This case-control study was performed on 75 women with preterm delivery (case group) and 75 women with term delivery (control group). The research tools included a questionnaire, polymerase chain reaction (PCR) assay of cervical swab samples and ELISA assay of umbilical cord blood samples. Fisher’s exact test and t test were also performed to compare qualitative variables between the two groups.

**Results:**

In this study, the mean age of subjects was 26.55 ± 0.53 years in the control group and 26.76 ± 0.56 years in the case group. The prevalence of *C. trachomatis* in the cervical swab samples was 7 (9.33%) in the control group and 2 (2.67%) in the case group. There was no *C. trachomatis* IgM antibody in either of the groups, while there was 1 (1.33%) *C. trachomatis* IgG antibody in both groups.

**Conclusion:**

The results of the present study showed that there was no significant relationship between *C. trachomatis* infection and preterm delivery.

## INTRODUCTION

Preterm delivery is an important subject in gynecology, obstetrics and pediatrics (1). Care and treatment of congenital complications in premature infants can be costly and accompanied by trauma and negative psychological impacts on the families. Generally, the birth of a term, healthy infant is the main goal of any pregnancy (2, 3). On the other hand, preterm delivery is defined as regular uterine contractions every five to eight minutes or less, lasting for 30 seconds. It is associated with progressive changes in the cervix, resulting in delivery after 22 weeks and before 37 weeks of gestation (2, 3).

Research shows that different factors play a role in preterm delivery, including urinary tract infections (UTIs), diabetes mellitus, renal diseases, cardiac diseases, multiple birth and recurrent pregnancy loss, among which UTIs account for 25–40% of all cases (4). Evidence shows that chronic infection and inflammation are among the primary causes of preterm delivery (5). Also, intrauterine and genital tract infections, which may result in the rupture of membranes, seem to contribute to preterm delivery (6, 7).

*Chlamydia trachomatis* (CT) is a small, Gram-negative, immobile coccoid, which lives as an obligate parasite inside human and animal cells. However, a large percentage of patients with CT are asymptomatic (8, 9). It has been shown that CT infection is one of the most prevalent sexually transmitted diseases (STDs) worldwide (2). There have been multiple studies on the role of CT infection in preterm delivery. Some of these studies have mentioned its role in preterm delivery, while some have ruled it out as an influential factor (10–13). Therefore, in the present study, we aimed to perform a more precise analysis of the role of CT infection in preterm delivery by examining women’s cervical swabs and umbilical cord blood samples.

## MATERIALS AND METHODS

The present study was performed on 75 women with preterm delivery (after 24 weeks of gestation and before 36 weeks and six days) as the case group and 75 women with term delivery as the control group. The participants were in the age range of 14 to 40 years and were referred to Besat Hospital of Sanandaj, Iran, in 2018–2019. Informed consent was obtained from all of the participants, and the study was ethically approved (IR.MUK.REC1395.106). The inclusion criteria were being sexually active (having sex during pregnancy) and non-consumption of antibiotics one week before sampling. On the other hand, the exclusion criteria were as follows: having a multiple pregnancy (i.e., a woman completing two or more pregnancies to 20 weeks of gestation or more); high blood pressure (BP≥140/90 mmHg); diabetes mellitus (FBS≥126 or HbA1C>6.5 or random BS>200); and being immunocompromised, based on the patient’s history (e.g., chemotherapy, radiotherapy, malnutrition, current treatment with corticosteroids, cancer, abnormal CBC differential and positive HIV test).

The research tools included a questionnaire, polymerase chain reaction (PCR) assay of cervical swab samples and ELISA assay of umbilical cord blood samples. At first, written consent forms were obtained from the participants, and then, the questionnaire was completed by a midwife. After completing the questionnaires, two cervical swab samples (one for freezing and one for extraction) and one umbilical cord blood sample were taken from each participant.

The cervical swab samples were immediately placed in 15-mL tubes, containing 5 mL of phosphate-buffered saline (PBS) and kept at −20 ± 2°C until DNA extraction. The blood samples were transferred to a −70 ± 2°C refrigerator. After collection of all cervical swab samples, DNA extraction was performed, using a High Pure PCR Template Preparation Kit (Roche, Germany). After extraction, a PCR assay was conducted, using the designed primers in a final volume of 25 μL. Next, the ELISA assay was performed on umbilical cord blood samples for detection of IgM and IgG antibodies against CT, using an Abnova CT Kit (Taoyuan, Taiwan).

### PCR assay.

Specific primers were designed for *16srRNA* gene of CT genome from the GenBank. The primer sequences were as follows: Forward: 5′-TGGCGGCGT GGATGA GGCAT-3′; and reverse: 5′-CTC AGT CCC AGT GTT GGC GG-3′. The length of the PCR target was 300 bp. The PCR reactions were performed in a total volume of 25 μL, containing PCR Master Mix.

### PCR amplification.

The PCR amplification program was as follows: Initial denaturation at 94°C for five minutes, followed by 30 cycles of denaturation at 94°C for 30 seconds, annealing at 64°C for 30 seconds, extension at 72°C for 25 seconds; and final extension at 72°C for five minutes. The PCR products were separated by electrophoresis on 1.5% agarose gel, stained with ethidium bromide, and visualized under ultraviolet (UV) light. The CT serovar L2 (strain 434/Bu/ATCC VR-902B) was also used as the PCR positive control.

### Statistical analysis.

Data were entered in SPSS version 20 and presented as percentage and mean in tables and diagrams. Quantitative values were stated as mean ± standard deviation. Fisher’s exact test, *t* test, and Chi-square test were used to compare qualitative variables between the two groups. P<0.05 was considered statistically significant.

## RESULTS

**Fig. 1.**
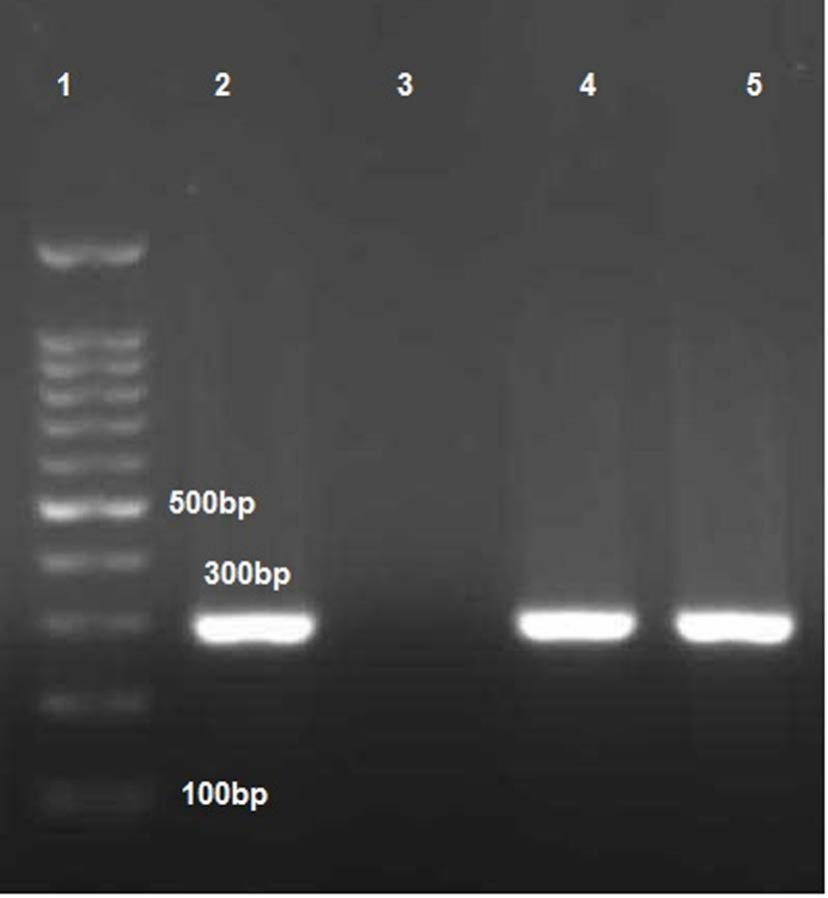
Polymerase chain reaction (PCR) assay for *Chlamydia trachomatis* (CT) detection. Lane 1: 100-bp DNA ladder (SinaClon, Tehran, Iran); lane 2: PCR-positive control (300 bp); Lane 3: negative control; Lanes 4–5: positive PCR products.

In this study, the mean age of the subjects was 26.55 ± 0.53 years in the control group and 26.76 ± 0.56 years in the case group. The highest educational level was high-school diploma in both the control (n=55, 73.33%) and case (n=40, 53.33%) groups. The mean age of the last pregnancy was 39.43 ± 0.1 years in the control group and 34.56 ± 0.2 years in the case group. The prevalence of CT in the cervical swab samples was 7 (9.33%) in the control group and 2 (2.67%) in the case group. The prevalence of CT IgM antibodies was zero in both groups, while the number of CT IgG antibodies was 1 (1.33%) in both groups. In one sample, both cervical swab samples and IgG titer were positive (Table 1). The PCR results and band patterns are presented in Fig. 1.

**Table 1.** Distribution of demographic data, history of risk factors, and prevalence of *Chlamydia trachomatis* (CT) infection in women with term delivery (control) and women with preterm delivery (case)

**Variables**	**Control (n=75)**	**Case (n=75)**	**P-value^1^**
Age, mean ± SD (years)	26.55 ± 0.53	26.76 ± 0.56	0.78
Age of last pregnancy, mean ± SD (years)	39.43 ± 0.1	34.56 ± 0.2	0.00
Educational level:			
Illiteracy or primary school education	11 (14.67%)	26 (34.67%)	
Middle school to high-school diploma	55 (73.33%)	40 (53.33%)	0.01
Academic degree	9 (12%)	9 (12%)	
Place of residence:			
The capital of the Province	74 (99%)	66 (88%)	
Other cities in the Province	1 (1%)	9 (12%)	0.01
CT	7 (9.33%)	2 (2.67%)	0.16
Parity:			
≥1	32 (42.67%)	21 (28%)	
0	43 (57.33%)	54 (72%)	0.06
IgG antibody against CT	1 (1.33%)	1 (1.33%)	1.00
History of vaginal infection	10 (13.33%)	7 (9.33%)	0.43
History of urinary infection	14 (18.67%)	7 (9.33%)	0.09
History of urinary infection and spouse	5 (6.67%)	7 (9.33%)	0.54
Preventive methods:			
Oral contraceptive pills	6 (8%)	10 (13.33%)	
Withdrawal	57 (76%)	55 (73.33%)	0.44
Intrauterine device	10 (13.33%)	6 (8%)	
Barrier methods	2 (2.67%)	4 (5.33%)	

Parity is determined by the number of pregnancies reaching 20 weeks of gestation (parity).

## DISCUSSION

STDs are highly prevalent infectious diseases in different countries, imposing a heavy burden on both communities and patients (2). The global spread of STDs resembles the spread of human immunodeficiency virus (HIV) infection. It has been shown that STDs, with or without lesions, increase the risk of infection with HIV. The persistence of an STD is a major public health concern in many countries. Therefore, STD management is one of the most important aspects of the control and treatment of HIV infection, especially in regions with high rates of STDs (14).

CT is among common factors contributing to STDs. This bacterium infects the epithelial cells of the cervix, as well as female and male urethras (4, 15). However, most of these infections are neither diagnosed nor treated, especially in women, resulting in pelvic inflammatory disease, ectopic pregnancy, or infertility (4, 15). Among these infections, the role of CT in preterm delivery has been discussed in various studies (4). In this regard, Seong et al. (2012) conducted a study in Seoul, South Korea, on 126 females with preterm delivery to understand the role of genital infections, using culture and PCR studies. They found that the prevalence of CT infection was approximately 2.4%. Similar to our results, there was no significant relationship between chlamydial infection and preterm delivery. The importance of our analysis is due to the concurrent assessment of mothers and neonates, although the sample size of the mentioned study was large (10).

Moreover, in a case-control study by Selveria et al. (2009) on 2127 women, the prevalence of CT infection was estimated at 4.7%. Although the sample size of this study was very large, there was no significant relationship between chlamydial infection and preterm delivery, which is similar to our results (16). Also, Schmidt et al. (2015) performed a cross-sectional study of 1452 deliveries. After studying the urine samples by a molecular method, the prevalence of CT infection was measured to be 13.9% among 378 (26%) women with preterm delivery; moreover, they suggested CT screening tests. Unlike our study, urine samples were used, whereas cervical swabs were not sampled in our study, and UTIs were excluded (17).

In another case-control study by Bogavec (2000) on 53 women with preterm delivery and 63 women with normal delivery, direct immunofluorescence (DIF) showed that the prevalence rates of CT infection were 4.3% and 11.3% in the case and control groups, respectively; therefore, they suggested a CT screening test during pregnancy. It should be noted that in their study, contrary to our study, the role of chlamydia infection in preterm delivery was described, and the DIF method was used (18). Also, in a study by Mikhova et al. (2007), the role of infection during pregnancy was investigated among 48 patients with term delivery and 40 women with delivery between 24 and 37 weeks of gestation to detect CT infection, using PCR assays. CT was detected in 5% of preterm deliveries and 2.08% of term deliveries. Similar to our results, there was no significant relationship between chlamydia infection and preterm delivery; however, diagnostic tests were recommended during pregnancy (19).

In a meta-analysis by Ahmadi et al. (2018), the relationship between chlamydia infection and preterm delivery was significant. The difference between the results of this study and our study is the simultaneous analysis of infection in the mother and neonate, as well as the use of two different methods for assessments. It should be noted that the results of this meta-analysis were based on the findings of previous studies on different samples, using different methods (20). Other studies have reported different results regarding the relationship between CT and preterm delivery (21–23).

In most previous studies, female cervical swabs or urine samples were used for the detection of CT infection, whereas no simultaneous sampling was performed on umbilical cord blood samples and amniotic fluid to determine the role of this infection in preterm delivery. However, in the present study, contrary to most previous research, to investigate the role of CT in preterm delivery, cervical swabs and umbilical cord blood samples of the placenta were used simultaneously, and diagnoses were made using both samples.

Based on the present results, although CT infection was detected in the cervical swab samples, IgM antibodies were indicators of active infection, formed by the fetus when infected. However, they were not present in the umbilical cord blood samples. Also, IgG antibodies, which were found in both groups, could be related to the mother’s prior infections or transferred from the placenta; however, there was no active infection caused by CT during pregnancy. Therefore, concurrent cervical and umbilical cord blood sampling may indicate that this infection cannot pass through the vaginal canal to infect the placenta or the embryonic membrane, which can lead to inflammation and rupture of the amniotic sac, associated with preterm delivery.

It is recommended that future studies investigate the role of other infections in terms of inflammation in the placenta and the embryonic membrane, using amniocentesis, which is the pathological study of the placenta and the embryonic membrane. The main limitation of this study was the lack of assessment of other STDs and women with sexual partners.

## CONCLUSION

The results of this study on women with preterm delivery, referred to Sanandaj Besat Hospital (Iran) during 2018–2019, showed that there was no significant relationship between preterm delivery and CT infection, according to both PCR and ELISA methods. Also, the main limitation of this study was the lack of infection studies in women with sexual partners, considering the cultural conditions of the region and the country.
